# Imaging of post-synaptic membrane trafficking in neuronal dendrites: progress, limitations, and new developments

**DOI:** 10.1117/1.NPh.10.4.044404

**Published:** 2023-08-04

**Authors:** David Perrais, Silvia Sposini, Julie Angibaud

**Affiliations:** University of Bordeaux, Interdisciplinary Institute for Neuroscience, CNRS, Bordeaux, France

**Keywords:** endocytosis, exocytosis, live cell fluorescence imaging, membrane trafficking, synaptic plasticity

## Abstract

Membrane trafficking of post-synaptic cargo is a key determinant of synaptic transmission and synaptic plasticity. We describe here the latest developments in visualizing individual exocytosis and endocytosis events in neurons using pH-sensitive tags. We show how these tools help decipher the spatial and temporal regulation of membrane trafficking steps during synaptic plasticity.

## 1 Introduction

**Fig. 1:**
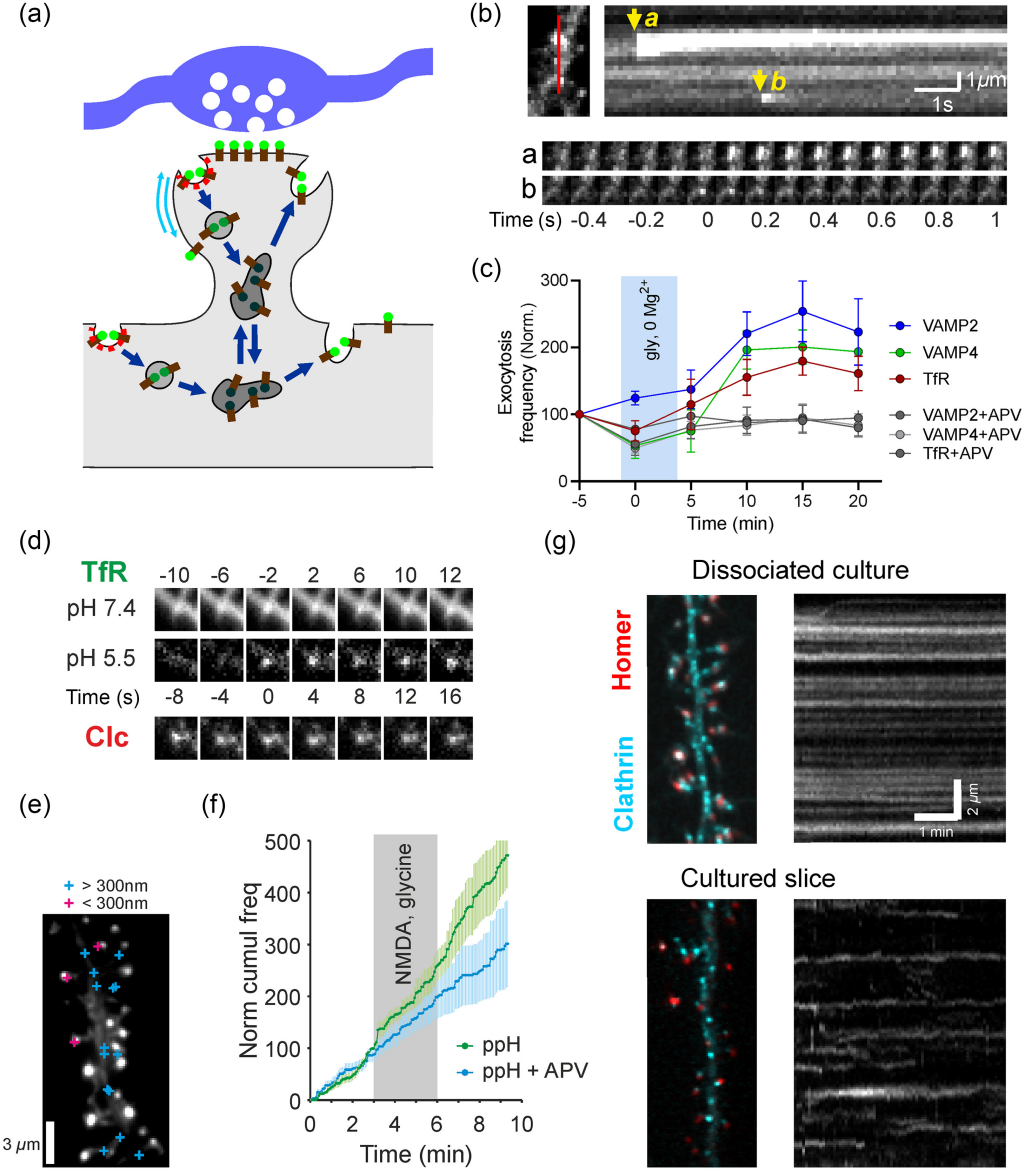
Visualization of post-synaptic membrane trafficking in neurons. (a) Scheme of the steps of membrane trafficking visualized with SEP-labeled cargo (brown sticks), visible at neutral pH (green lollipops) but not at the acidic pH of REs (dark gray). A presynaptic terminal is depicted in blue, and the PSD facing this axon in the post-synaptic spine has concentrated receptors. The internalization of receptors occurs near the PSD (red stippled line, figuring clathrin) or further away in the dendritic shaft. Blue arrows depict the progression of trafficking along the endosomal pathway to end in exocytosis near the PSD or further away. (b) Kymograph showing the detection of two exocytosis events in a portion of dendrite of a neuron transfected with TfR-SEP. For the event a, the vesicle is visible for several seconds, due to kiss-and-run exocytosis, while for event b, it is transient due to receptor diffusion. Reprinted from Ref. 8. (c) The frequency of exocytosis events, visualized by VAMP2-SEP, VAMP4-SEP, or TfR-SEP, increases after induction of LTP by the perfusion of a solution containing glycine (100  μM) and no ${\mathrm{Mg}}^{2+}$, which activates synaptic NMDA receptors. Reprinted from Ref. 9. (d) Images of a portion of dendrite taken at alternating pH of 7.4 and 5.5 enable the detection of an endocytic vesicle containing TfR-SEP at time 0. It appears at a CCS labeled with clathrin. (e) Location of endocytic events (blue and magenta crosses) relative to PSDs labeled with Homer1c-tdTomato (image of the fluorescent label). (f) Cumulative frequency of endocytic events labeled with SEP-GluA2. It increases during induction of LTD with NMDA. (d)–(f) Reprinted from Ref. 10. (g) Images of a cultured neuron (top) and a CA1 pyramidal neuron in a cultured hippocampal slice (bottom) transfected with Homer1c-tdTomato and clathrin-GFP. In both cases, CCS are visible throughout the dendritic shaft and in most spines. Right, kymographs of clathrin-GFP show that they are very stable in cultured neurons but transient in the slice.

Membrane trafficking in dendrites, in particular exocytosis, endocytosis, and recycling of post-synaptic receptors, is a key determinant of synaptic transmission and synaptic plasticity.^1^ Indeed, blocking exocytosis mediated by VAMP1-3 with post-synaptic dialysis of tetanus toxin, through a patch-clamp recording pipette, bocks long-term potentiation (LTP)^2^^–^^4^ while blocking dynamin mediated endocytosis with a peptide interfering with its function blocks long-term depression (LTD).^5^ In addition, exocytosis of recycling endosomes (REs) increases following LTP induction^6^ and endocytosis of post-synaptic receptors increases following LTD induction.^7^ Based on these data, a model has been built describing endocytosis, sorting and recycling of post-synaptic receptors within dendrites [Fig. 1(a)]. However, its spatial organization and dynamics, as well as many molecular players involved are still unknown. Therefore, live cell imaging of individual exocytic or endocytic events is crucial to determine if and how membrane trafficking is modulated following the induction of synaptic plasticity, and whether these processes contribute to its spatial selectivity.^11^ Here we will review the methods developed to image individual exocytosis and endocytosis events, how it helped decipher the cellular and molecular mechanisms, and the challenges ahead to deal with their limitations.

## 2 Imaging of Individual Exocytosis Events

The method of choice to image exocytosis events has been to rely on the fact that intraluminal pH of secretory vesicles, recycling endosomes (REs), or synaptic vesicles is acidic (pH ∼5.5 to 6). Therefore, the fast change in pH (from 5.5 to extracellular pH 7.4) occurring at the time of exocytosis can be detected by a pH-sensitive fluorophore conveniently positioned at the intraluminal/extracellular side of the transmembrane cargo of interest, such as post-synaptic α-amino-3-hydroxy-5-methyl-4-isoxazolepropionic acid receptors (AMPARs), or the transferrin receptor (TfR), a ubiquitous RE marker. The pH-sensitive protein with the close to ideal properties to sense this transition is the GFP mutant super-ecliptic pHluorin (SEP), isolated more than 20 years ago.^12^ The SEP is virtually non-fluorescent at pH 5.5, making the SEP-labeled cargo in REs or secretory vesicles invisible, such that single exocytosis events can be visualized as bright punctae throughout somatodendritic compartments of the neuron, including dendritic spines. Modeling of exocytosis and diffusion in the plasma membrane with experimentally derived parameters shows that AMPAR exocytosis must occur in the vicinity of synapses for rapid control of AMPAR number at synapses.^13^ After exocytosis, fluorescence decays with various kinetics, revealing different modes of exocytosis [Fig. 1(b)].^8^^,^^14^^,^^15^ Photobleaching of parts, or even the whole cell, nearly erases the fluorescence of fluorescent of cargo residing in the plasma membrane while preserving the non-fluorescent cargo in acidic compartments, allowing a better isolation of exocytic events and quantification of whole-cell exocytosis rates.^9^^,^^16^ In addition, the red pH-sensitive fluorescent proteins, pHuji and pHmScarlet^17^^,^^18^ or SNAPtag ligands labeled with the pH-sensitive red fluorophore Virginia Orange,^19^ are available to permit multicolor imaging of various cargo proteins. Recent developments in research on post-synaptic exocytosis and synaptic plasticity include the effect of local induction of LTP at single spines on exocytosis,^20^^–^^22^ the role of L-type calcium channels in controlling exocytosis after LTP induction,^22^ and the identification of several classes of REs containing either VAMP2 or VAMP4 having distinct roles in LTP [Fig. 1(c)].^9^

## 3 Imaging of Individual Endocytosis Events: Clathrin Dynamics

Unlike exocytosis, the formation of endocytic vesicles is not accompanied by sudden changes in pH, rendering imaging of endocytosis at high speed with single-vesicle resolution difficult.^23^ In the case of clathrin-mediated endocytosis (CME), one solution is to label clathrin or associated proteins and image clathrin-coated structures (CCSs). The CCSs are located all over dendrites, but a significant proportion is located in the vicinity of post-synaptic densities (PSD), <300  nm away, such that 75% to 85% of PSDs have a nearby CCS.^10^^,^^24^^–^^26^ The proportion of PSDs bearing a CCS is decreased to ∼40% when the three isoforms of Shank1-3, PSD proteins that interact with endocytic and actin binding proteins, are downregulated by a miRNA^27^ or when the immediate early gene Homer1a is overexpressed, which displaces Shank proteins from PSDs.^25^ Peri-PSD CCSs play a specific role in post-synaptic receptor endocytosis,^25^ even if some AMPARs may internalize through clathrin independent endocytosis.^28^^–^^30^ How does imaging of CCSs reveal the dynamics of CME? The CCSs are transient structures appearing and disappearing in living cells: this would reflect the clustering of cargo, invagination to form a vesicle, scission, clathrin uncoating, and movement away from the plasma membrane. Therefore, CCS lifetime, around 1-2 min in most cell types, can be used as a proxy for endocytic activity with single CCS resolution.^31^ However, super-resolution microscopy and correlative light electron microscopy have revealed the existence of complex CCSs that produce more than one endocytic vesicle.^32^^,^^33^ Therefore, vesicles can form without CCS disappearance or even measurable changes observed with classical wide field microscopy, so-called non-terminal endocytic events (Refs. 34 and 35; see also following paragraph). In dendrites of mature neurons in culture, CCSs are almost all stable in a period of at least 10 min, despite the fact that they internalize CME cargo at a much higher rate.^10^^,^^24^^–^^26^ Therefore, even if some changes in the size or number of CCS have been observed after induction of LTP or LTD,^26^ observing CCSs in living neurons cannot reveal the precise moment of vesicle formation, hence the rate of endocytosis.

## 4 Imaging of Individual Endocytosis Events: Direct Detection of Vesicle Formation

By definition, endocytosis depicts the process of vesicle formation, i.e., the isolation of a membrane cargo from the extracellular space. Testing this connection could thus provide a direct assay for vesicle formation, overcoming the limitation of observing CCS dynamics. This connection can be tested with a cargo protein labeled with pH-sensitive tag such as SEP and with repeated pulsed pH changes (ppH) from 7.4 to 5.5. Cargo on the plasma membrane becomes invisible at extracellular pH 5.5 while cargo in non-acidic vesicles (e.g., endocytic vesicles not yet acidified) will remain visible. The moment of vesicle formation is thus detected when a pH resistant vesicle appears in an image taken at extracellular pH 5.5, with a temporal precision matching the ability to exchange the two solutions rapidly, typically less than 2 s for whole cells but faster for small neurites [Fig. 1(d)].^8^ A good cargo protein to use is, like for RE exocytosis, TfR-SEP. In addition to its location in RE (which are acidic and thus invisible with the SEP tag), it is also located on the plasma membrane and constitutively internalized through CME. Multiple control experiments show that CME is not affected during the ppH protocol.^34^^,^^35^ In neurons, despite the activation of acid-sensing ion channels by the low pH solution, blocking these channels does not affect the rate of CME, and quenching SEP fluorescence with cell-impermeable Trypan purple instead of acidic pH enables the detection of vesicles, albeit for only a few minutes due to progressive accumulation of the dye on the plasma membrane.^10^ Multiple endocytic events indeed appear at individual CCSs with a median inter-event interval of 168 s.^10^ Moreover, endocytic vesicles containing the AMPAR subunit SEP-GluA2 detected with the ppH protocol form preferentially near PSDs while those containing the non-synaptic TfR-SEP do not [Fig. 1(e)].^10^ Moreover, application of *N*-methyl-D-aspartic acid (NMDA), which leads to internalization of AMPARs and LTD, provokes a transient increase in the frequency of SEP-GluA1 and SEP-GluA2 endocytic events [Fig. 1(f)].^10^^,^^29^^,^^36^^,^^37^ The characterization of the dynamics of post-synaptic endocytosis is thus well under way. Nevertheless, several outstanding questions remain. How is endocytosis regulated after LTD at individual spines? What is the fate of endocytic vesicles? Are specific populations targeted to degradation or recycled? Does it occur locally? To address these questions at the single event level, new protocols and tools are required with new types of markers, such as protocols, inducing LTD in individual spines^38^ or fluorophores with inverse pH sensitivity.^39^

## 5 Current Limitations and Perspectives

Endogenous AMPARs are for the most part heteromers of GluA subunits associated with several accessory proteins,^40^ so overexpression of SEP-GluA subunits likely biases the labeling towards homomeric receptors with specific trafficking routes.^30^ Genome editing of GluA genes to tag endogenous AMPARs, either in single neurons^41^^,^^42^ or in transgenic mice with bi-allelic gene editing^43^ should reveal the unbiased trafficking of post-synaptic receptors. Another important step towards understanding membrane trafficking in a physiological context is the ability to image tagged proteins of interest in more intact systems. Although most live imaging studies have been performed in cultured neurons grown on glass coverslips, imaging of exocytosis in cultured slices has been proven possible by two photon microscopy.^20^ Imaging endocytosis in slices remains more challenging, as the ppH-based methods may lack the required time resolution due to the convoluted extracellular space. Nevertheless, our preliminary data show that CCSs are more dynamic in slices than in cultured neurons on glass [Fig. 1(g)], paving the way for an estimation of local endocytic activity and its modulation during synaptic plasticity based on imaging of CCSs.

Finally, to better assess the membrane trafficking of receptors in a physiological context *in situ*, we can anticipate the development of brighter pH-sensitive fluorescent proteins (it is remarkable that SEP was not optimized since its discovery in 2000, despite the constant improvement of GFP based fluorescent proteins^44^) and fast, sensitive imaging techniques, such as lattice light-sheet microscopy with adaptive optics.^45^
